# Systemic BCG Immunization Induces Persistent Lung Mucosal Multifunctional CD4 T_EM_ Cells which Expand Following Virulent Mycobacterial Challenge

**DOI:** 10.1371/journal.pone.0021566

**Published:** 2011-06-24

**Authors:** Daryan A. Kaveh, Véronique S. Bachy, R. Glyn Hewinson, Philip J. Hogarth

**Affiliations:** TB Research Group, Animal Health and Veterinary Laboratories Agency (AHVLA), Addlestone, Surrey, United Kingdom; Institut Pasteur, France

## Abstract

To more closely understand the mechanisms of how BCG vaccination confers immunity would help to rationally design improved tuberculosis vaccines that are urgently required. Given the established central role of CD4 T cells in BCG induced immunity, we sought to characterise the generation of memory CD4 T cell responses to BCG vaccination and *M. bovis* infection in a murine challenge model. We demonstrate that a single systemic BCG vaccination induces distinct systemic and mucosal populations of T effector memory (T_EM_) cells in vaccinated mice. These CD4^+^CD44^hi^CD62L^lo^CD27^−^ T cells concomitantly produce IFN-γ and TNF-α, or IFN-γ, IL-2 and TNF-α and have a higher cytokine median fluorescence intensity MFI or ‘quality of response’ than single cytokine producing cells. These cells are maintained for long periods (>16 months) in BCG protected mice, maintaining a vaccine–specific functionality. Following virulent mycobacterial challenge, these cells underwent significant expansion in the lungs and are, therefore, strongly associated with protection against *M. bovis* challenge. Our data demonstrate that a persistent mucosal population of T_EM_ cells can be induced by parenteral immunization, a feature only previously associated with mucosal immunization routes; and that these multifunctional T_EM_ cells are strongly associated with protection. We propose that these cells mediate protective immunity, and that vaccines designed to increase the number of relevant antigen-specific T_EM_ in the lung may represent a new generation of TB vaccines.

## Introduction

Tuberculosis (TB) caused by infection with *Mycobacterium tuberculosis* or *Mycobacterium bovis* remains one of the most important infectious diseases of man and animals respectively, and continues to inflict a huge cost in humans and animals in both health and financial terms [1].

At present the only available vaccine against TB is *M. bovis* bacille Calmette-Guérin (BCG) which demonstrates variable efficacy in humans and cattle [2], [3]. In particular, BCG induces effective protection against childhood disseminated TB and tuberculous meningitis, but poor protection against pulmonary TB in adolescents and adults [4]. Despite this inconsistent performance, BCG remains the most widely used human vaccine in the world and due to its partial efficacy and proven safety record, is unlikely to be withdrawn. Hence, a great deal of research effort is targeted toward improving the efficacy of BCG by a number of approaches; prominent among which is boosting BCG with heterologous vaccines [5].

A deeper understanding of the T cell mechanisms underpinning the immunity induced by BCG vaccination would help to identify immune correlates of protection. This would facilitate and accelerate the rational design of improved replacement, or adjunct tuberculosis vaccines.

BCG induced protection is dominated by antigen-specific CD4 T cells with a lesser and as yet poorly defined contribution by CD8 T cells. IFN-γ is essential and combined with TNF-α production considered to play a central role in protection [6]. Cryptically, measurement of circulating antigen-specific CD4 T cell production of these cytokines fail to offer reliable a correlate of protection in many studies [7], [8]; whilst they do correlate in others [9], [10], [11] reviewed by Goldsack & Kirman [12]. These data then indicate that whilst CD4 T cell generated IFN-γ production is an essential component of the protective response; it does not effectively describe all of the mechanisms acting toward successful protective immune responses. This is further demonstrated by studies in which, despite increasing the frequency of IFN-γ producing CD4 T cells protective immunity was not increased [13], [14].

The recent identification and description of multifunctional T cells may represent another major essential component in BCG induced protection. Multifunctional CD8 T cells associate with non-progressors in HIV infection [15], multifunctional CD4 and CD8 T cells characterise protective immunity in the lungs of influenza infected mice (reviewed by Kohlmeier and Woodland, [16]). Multifunctional CD4 T cells have been identified, and represent a correlate of protection in a murine leishmania vaccination/challenge model [17]. More recently, they have been described in *M. tuberculosis* infection and vaccination models in mice [18], [19], non-human primates (NHP) [20] and human vaccination studies [17], [21]. These studies show a distinct correlation between the frequencies of these cells and the expression of protective immunity. Despite these encouraging results, however, formal demonstration that these cells mediate protection during vaccination against tuberculosis remains elusive. In studies of human infection however, the role of multifunctional T cells remains more cryptic and they may represent a correlation with active disease [22], [23], [24].

We sought to investigate the role of these cell types in our previously established murine BCG vaccination and *M. bovis* challenge model [25]. Importantly, we were able to demonstrate that intradermal, non-mucosal, BCG immunization was able to induce a persistent, long lived population of multifunctional CD4^+^ cells in the lungs which preferentially expand following challenge. These cells exhibited a complex effector memory (T_EM_) phenotype and associate with BCG induced protection against mucosal tuberculosis challenge.

Our study is the first to describe significant expansion of BCG-specific lung resident multifunctional CD4 T_EM_ cells during the protective response to virulent mycobacterial challenge. We propose that these cells mediate protective immunity, and that vaccines designed to increase the number of relevant antigen-specific T_EM_ in the lung may represent a new generation of TB vaccines.

## Results

### BCG immunization induces significant protection in spleen and lung against *M. bovis* challenge

To establish the efficacy of BCG to induce protection against *M. bovis* intranasal (i.n.) challenge, two separate duration experiments were undertaken. First, groups of BALB/c mice were BCG or sham immunized by the intradermal route (i.d.) six weeks prior to i.n. challenge with *M. bovis*. Upon determination of bacterial load four weeks after challenge, BCG immunised groups displayed highly significant protection in the spleen ∼1.5 log_10_ (ρ<0.001) and lungs ∼2 log_10_ (ρ<0.01) (Table 1 ^A^, representative data of 1 of 4 separate experiments).

**Table 1:** BCG induced protection in the spleen and lungs of *M. bovis* challenged mice.

	Spleen			Lung		
	Log_10_ CFU	S.E.	Log_10_ protection	Log_10_ CFU	S.E.	Log_10_ protection
**Experiment 1** a						
**Control**	4.86	0.17	-	6.95	0.30	-
**BCG**	3.38	0.10	1.48 ***	5.07	0.14	1.88 **
**Experiment 2** b						
**Control**	4.16	0.23	-	6.18	0.10	-
**BCG 6 wks**	3.04	0.21	1.12**	5.07	0.11	1.11***
**BCG 1 year**	2.62	0.10	1.55***	4.81	0.18	1.37***

a Experiment 1, mice immunized 6 weeks prior to challenge: un-paired student's two-sided *t* - test ** ρ<0.01, *** ρ<0.001 (n = 5).

b Experiment 2, mice immunized six weeks or one year prior to challenge: one-way ANOVA with Bonferroni post-test * ρ<0.05, ** ρ<0.01, *** ρ<0.001 (n = 8).

Second, groups of mice were BCG or sham immunized as described at week 0 or BCG immunized at week 48 and all challenged at week 54. Again, BCG immunised groups displayed highly significant protection in the spleen and lungs (ρ<0.001) Table 1 ^B^.

### BCG induces a higher frequency of IFN-γ producing CD4 T cells in the lungs than spleen

**Figure 1:**
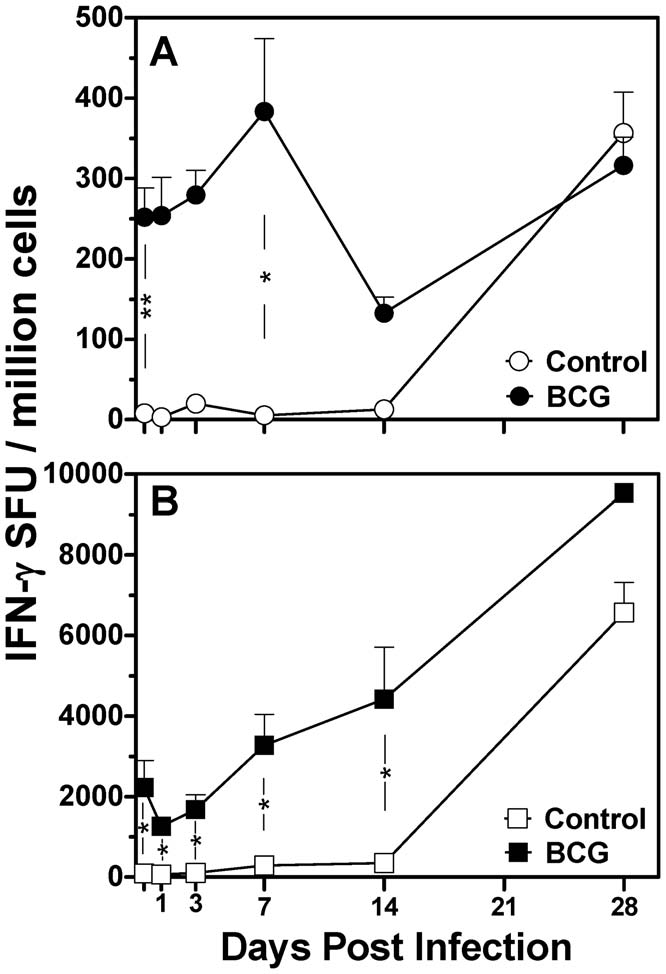
Frequency of IFN-γ secreting cells in spleen and lung from BCG immunized mice following *M. bovis* challenge. Lymphocytes of the spleen (A) and lungs (B) from mice (n = 5) immunized six weeks previously with BCG or placebo were isolated prior to and post challenge with *M. bovis*. Following re-stimulation with antigen cocktail, cells were enumerated for IFN-γ production by ELISPOT. Symbols represent mean (±S.E.) spot forming units adjusted per 10^6^ cells. Un-paired student's two-sided *t* - test * ρ<0.05, ** ρ<0.01.

Given the established importance for IFN-γ producing CD4 T cells in protection against tuberculosis infection, we assessed the frequency of antigen-specific IFN-γ cells by ELISPOT during the course of *M. bovis* infection in BCG or sham immunised animals. In the spleen (Figure 1A), immunization induced a mean frequency of 252 Spot Forming Units (SFU)/million cells compared to controls (8 SFU, ρ<0.01). Following challenge this increased transiently for the first seven days (383 SFU day 7, ρ<0.05 c.f. controls) and then declined to day 14. In the lung, however (Figure 1B), the pre-challenge frequency of IFN-γ producing cells in vaccinated mice was significantly higher than the spleen (2220 SFU/million cells, ρ<0.05). Following challenge, the frequency of IFN-γ producing cells in the vaccinated lung continued to increase (max. 9538 SFU) and was significantly higher than control mice at all time points except day 28. By 28 days post infection there were significant numbers of IFN-γ producing cells in the spleen (356 SFU) and the lungs (6570 SFU) of control animals, most likely indicating a response to *M. bovis* challenge rather than vaccination by this time. The frequency of IFN-γ producing cells was up to 30 fold higher in the lung than spleen during vaccination/infection, indicating that BCG induced a preferential mucosal rather than systemic antigen-specific immune response.

### BCG vaccination induces CD44^hi^ multifunctional CD4 T cells producing IFN-γ, IL-2 and TNF-α

**Figure 2:**
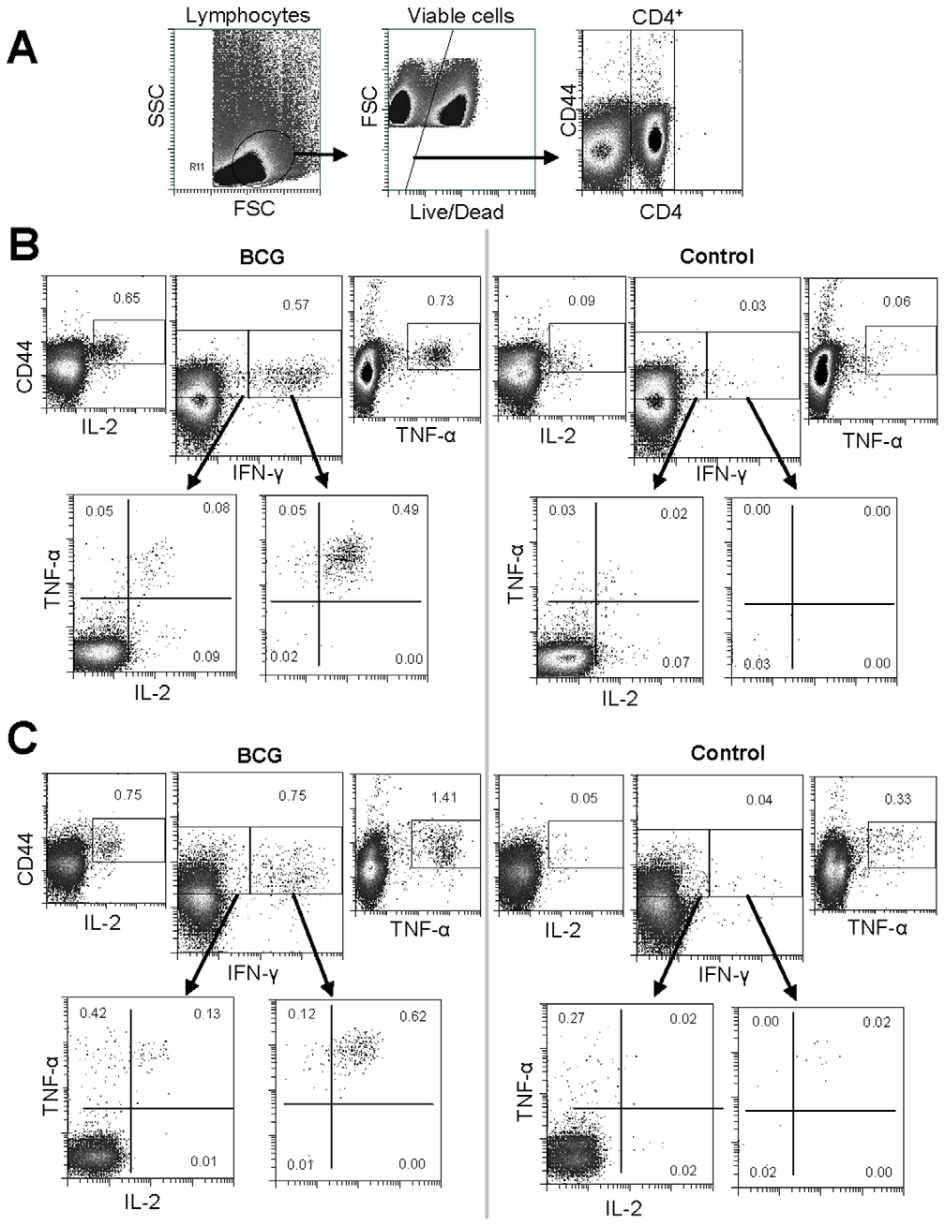
BCG induces CD4^+^CD44^hi^ T cells secreting IFN-γ, IL-2 & TNF-α. Six weeks following immunization, spleen (B) and lung (C) cells from BCG or sham control vaccinates were isolated, stimulated and stained by intracellular staining (ICS). Plots were gated on live CD4^+^ lymphocytes (A) and analyzed for all combinations of cytokine productivity. Numbers indicate percentage of CD4^+^ cells, data are representative of 1 of 3 independent experiments.

In order to determine the functionality of the responding T cells induced by BCG vaccination, spleen and lung lymphocytes were isolated from sham and BCG immunized animals at six weeks post vaccination. These cells were stimulated with a cocktail of mycobacterial proteins and subsequently interrogated by intracellular staining (ICS) and 9 colour flow cytometric analysis. Spleen cells derived from BCG vaccinated animals exhibited cytokine production by cells of a CD4^+^CD44^hi^ phenotype at a significantly greater frequency than cells derived from controls (IFN-γ, 0.57 vs. 0.03%; IL-2, 0.65 vs. 0.09%; TNF-α, 0.73 vs. 0.06%). CD4^+^ lung derived cells showed an equivalent frequency of cytokine producing cells (IFN-γ, 0.75 vs. 0.04%; IL-2, 0.75 vs. 0.05%; TNF-α, 4.41 vs. 0.33%) (Figure 2).

Further staining and analysis of these cytokine producing spleen and lung cells by ICS revealed the majority of them to be multifunctional, with 0.49% of CD4^+^ spleen cells and 0.62% of CD4^+^ lung cells being IFN-γ^+^IL-2^+^TNF-α^+^ (Figure 2).

### Multiple cytokine producing cells demonstrate a higher quality of response than single producers

**Figure 3:**
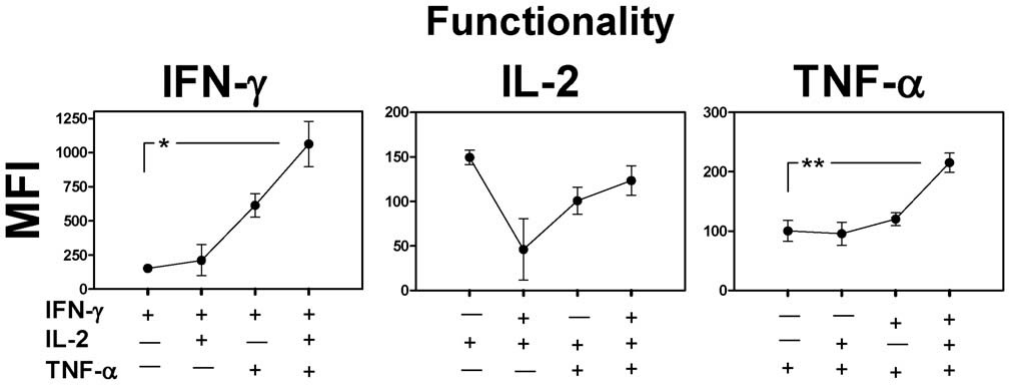
Multifunctional CD4^+^CD44^hi^ T cells produce a better quality of response. Six weeks following BCG immunization, spleen cells were isolated, stimulated and stained by ICS. The antigen-specific IFN-γ, IL-2 and TNF-α Median Fluorescence Intensity (MFI) of all 1^+^, 2^+^ or 3^+^ cytokine secreting subsets of CD4^+^CD44^hi^ cells were determined. Symbols represent the mean IFN-γ, IL-2 and TNF-α MFI of the indicated T cell phenotype. Un-paired student's two-sided *t* - test * ρ<0.05, ** ρ<0.01 (n = 4). Data are representative of 1 of 3 independent experiments.

Median Fluorescence Intensity (MFI) of cells analysed by ICS is a direct correlate of the physical amount of cytokine produced per cell. We measured the individual IFN-γ, IL-2 and TNF-α MFI of spleen cells at six weeks post vaccination in subsets of CD4^+^CD44^hi^ T cells producing the 7 potential cytokine combinations (Figure 3). For IFN-γ, the amount of cytokine produced increased with the number of cytokines being concomitantly expressed, from an IFN-γ MFI of 152 for IFN-γ single positive cells, to an MFI of 1063 for triple positive cells, an increase of 7 fold. For TNF-α production displayed a similar correlation with a TNF-α MFI of 100 for TNF-α single positive cells and an MFI of 215 for triple positive cells, a 2 fold increase. For IL-2, the MFI was highest in single positive cells (149), although these were not significantly greater than the triple positive cells (123). It should be noted that the overall lower MFI exhibited by IL-2 producing cells may lower the sensitivity of these measurements.

Thus, triple cytokine producing cells expressed a higher quality of response in terms of the amount of IFN-γ and TNF-α produced per cell.

### Multifunctional CD4 T cells express a CD44^hi^CD62L^lo^CCR7^−^CD27^−^ Phenotype

**Figure 4:**
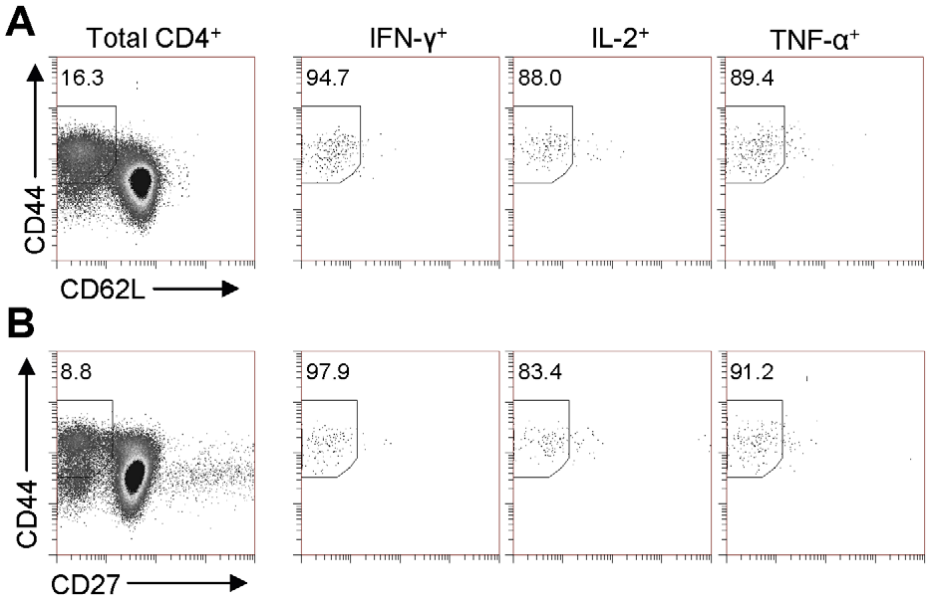
Multifunctional CD4^+^ cells have a CD44^hi^CD62L^lo^CD27^−ve^ phenotype. Spleen cells isolated from six week BCG immunized mice were stained for cytokine production by ICS and phenotyped for CD44, CD62L and CD27 expression. Regions represent % of cells exhibiting a CD4^+^CD44^hi^CD62L^lo^ (A) or CD4^+^CD44^hi^CD27^−^ (b) phenotype. Plots are gated on CD4^+^ cells and are representative of 1 of 3 independent experiments.

In order to establish whether the multifunctional cells induced by BCG immunization expressed a memory or effector type phenotype, we further investigated the surface phenotype of these cytokine producing cells in spleen (Figure 4) and lungs (data not shown) six weeks following vaccination. Gating on total CD4^+^ cells showed that 16% of these cells expressed a CD44^hi^CD62^lo^ T_EM_ phenotype and gating on the cytokine producing cells demonstrated that 95% of IFN-γ^+^, 88% of IL-2^+^ and 89% of TNF-α^+^ cells reside within this phenotype (Figure 4A). Staining for CCR7 expression revealed a negative phenotype, indicating that these cells were not central memory (data not shown). Further phenotyping revealed that whereas 9% of total CD4^+^ cells were CD44^hi^ and negative for CD27; 98% of IFN-γ^+^, 83% of IL-2^+^ and 91% of TNF-α^+^ cells displayed this surface phenotype (Figure 4B). The loss of CD27 appears to indicate that these cells have undergone terminal differentiation but still express memory markers. Together these data show that the cytokine producing cells express a CD4^+^CD44^hi^CD62L^lo^CCR7^−^CD27^−^ effector memory phenotype.

### IFN-γ producing cells in the lung reside in the lung interstitium compartment

In order to determine which compartment of the lung the IFN-γ producing cells reside in, lung interstitial cells and bronchoalveolar lavage (BAL) cells were isolated from BCG immunised mice. The frequency of antigen-specific IFN-γ cells in each cell population was assessed by ELISPOT.

**Figure 5:**
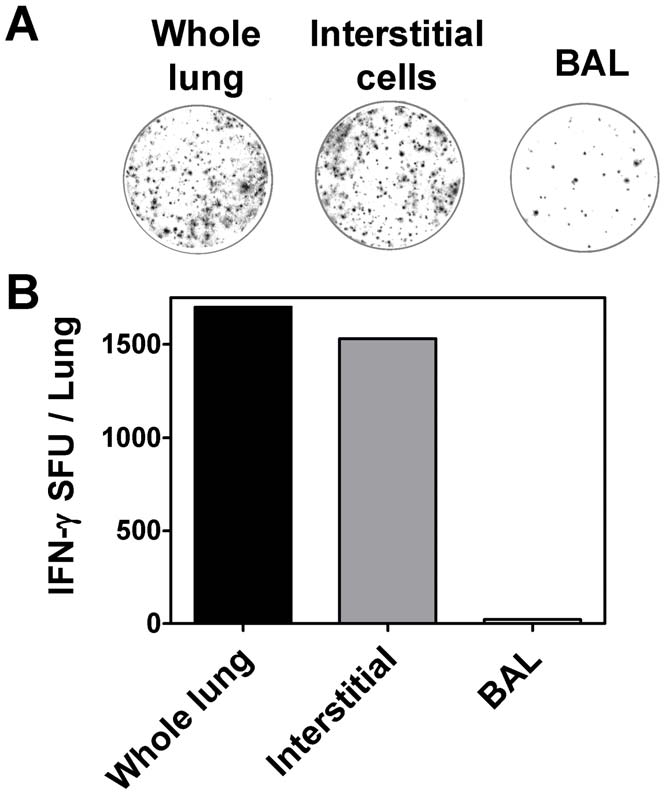
IFN-γ producing cells in the lungs reside in the lung interstitium compartment. Whole lung lymphocytes (without BAL performed), lung interstitial cells (after BAL performed) and bronchoalveolar lavage (BAL) cells were isolated from six week BCG immunized mice and assayed for IFN-γ production by ELISPOT. 5×10^5^ cells were cultured with antigen cocktail and developed by ELISPOT, photographs of representative wells (A), bars representing the mean (±S.E.) spot forming units (SFU) adjusted per lung (B). Representative example of 1 of 3 independent experiments.

Whole lung lymphocytes and lung interstitial cells prepared from BAL depleted lungs contained a similar frequency of IFN-γ secreting cells (1700 & 1500 SFU per lung), whereas BAL derived lymphocytes contained very few IFN-γ secreting cells (22 SFU per lung) (Figure 5). These results clearly demonstrate that the IFN-γ producing cells in the lungs induced by BCG vaccination reside within the lung interstitial cells and not the bronchoalveolar compartment.

### Multifunctional cells in the spleen express a lung homing phenotype

In order to investigate whether the antigen specific T cells identified in the lung are derived from the spleen; spleen and lung cells isolated from BCG and sham immunised mice were stained for the potential lung homing surface markers α1 integrin (CD49a), α4-integrin (CD49d), β1-integrin (CD29), CD11a and CXCR3.

**Figure 6:**
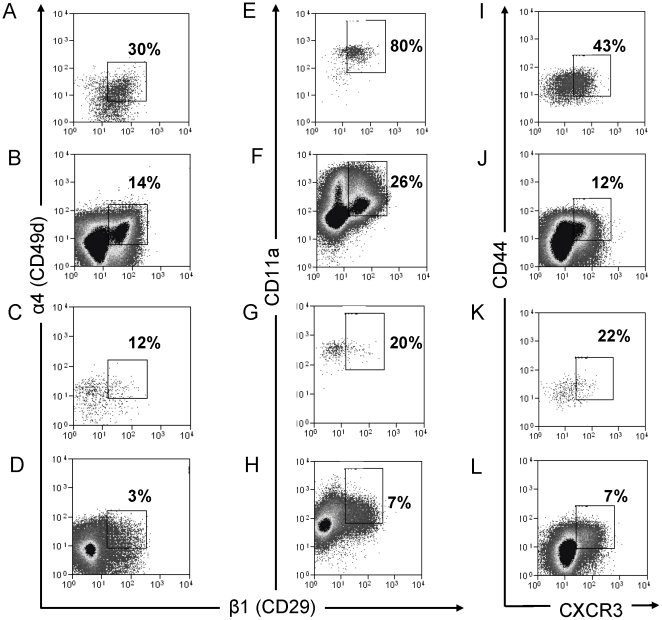
Multifunctional cells in the spleen express a lung homing phenotype. Spleen and lung cells isolated from six week BCG immunized mice were stained for cytokine production by ICS and phenotyped for homing markers. Plots are gated on CD4^+^ (or IFN-γ^+^CD4^+^) cells and regions represent % these cells exhibiting a positive homing marker phenotype for: α4-integrin (CD49d) and β1-integrin (CD29) by IFN-γ^+^CD4^+^ cells in the spleen (A) and the lung (C) or by the total CD4^+^ cell population in the spleen (B) and lung (D); CD11a by IFN-γ^+^CD4^+^ cells in the spleen (E) and the lung (G) or by the total CD4^+^ cell population in the spleen (F) and lung (H); and CXCR3 by IFN-γ^+^CD4^+^ cells in the spleen (I) and the lung (K) or by the total CD4^+^ cell population in the spleen (J) and lung (L). Plots are representative of 1 of 3 independent experiments.

Plots are shown for IFN-γ^+^ T cells (as we have shown the majority of IFN-γ^+^ cells are multifunctional for IL-2^+^ and TNF-α^+^), to overcome the limitations of low numbers of triple positive T cells recovered from the lungs in unchallenged mice (Figure 6).

CD4^+^CD44^hi^IFN-γ^+^ spleen cells were negative for the α1 integrin (CD49a) (data not shown), but 30% of IFN-γ^+^ cells were positive for α4β1 integrin heterodimer(Figure 6A) vs. only 14% of total CD4^+^ cells (6B). In the lung, this was reduced to 12% IFN-γ^+^ (6C) vs. 3% total CD4^+^ (6D).

80% of spleen IFN-γ^+^ cells co-expressed CD11a and β1 (Figure 6E) vs 26% total CD4^+^ (6F). In the lung this was reduced to 20% (6G) and 7% (6H), respectively.

High numbers of these IFN-γ^+^ cells also expressed high levels of the chemokine receptor CXCR3 (43% (6I) vs. 12% of total CD4^+^ (6J)). In the lung the trend for down regulation was maintained with 22% of IFN-γ^+^ cells expressing CXCR3 (6K) vs. 7% of total CD4^+^ cells (6L).

Together these data show that BCG induced multifunctional T cells in the spleen display up-regulated co-expression of the potential lung homing markers, α4β1 and CD11a, and up-regulated expression of the chemokine receptor CXCR3, which can confer lung homing properties to Th1 cells under inflammatory conditions (e.g. *M. bovis* infection). Conversely, analysis of these markers in lung derived BCG induced multifunctional T cells indicates a trend of down regulation, perhaps indicating that cells resident in the lung no longer need to express these markers.

### Vaccination induced, resident multifunctional CD4 T cells undergo preferential expansion in the lung following *M. bovis* intranasal challenge

**Figure 7:**
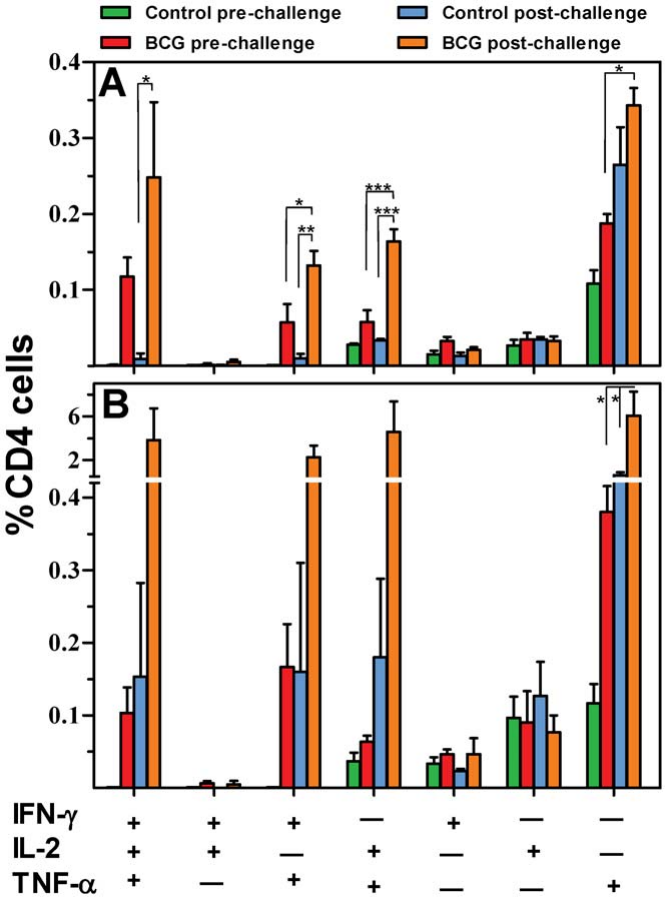
Vaccination induced resident multifunctional CD4^+^ cells in the spleen and lungs undergo expansion following challenge. The frequency of multifunctional CD4^+^CD44^hi^ cells was determined in the spleen (A) and lungs (B) of control mice, BCG immunized mice, and control and BCG immunized mice fourteen days post challenge. Bars represent mean (±SE) % frequency of cells of indicated T cell phenotype as a % of total CD4^+^ cells. Un-paired student's two-sided *t* - test * ρ<0.05, ** ρ<0.01, *** ρ<0.001.

Six weeks following vaccination, BCG and sham immunized mice were challenged with 600 CFU virulent *M. bovis* intranasally route. Spleen and lung lymphocytes were isolated prior to and 14 days following challenge and the frequencies of antigen-specific CD4^+^ CD44^hi^ cells exhibiting all seven combinations of IFN-γ, IL-2 and TNF-α production were determined (Figure 7). BCG immunization induced complex CD4 T cell responses in the spleen and lungs which increased in proportion following challenge.

In the spleen comprised: 0.11% IFN-γ^+^IL-2^+^TNF-α^+^ cells pre-challenge to 0.25% post-challenge,; 0.06% IFN-γ^+^TNF-α^+^ cells to 0.13%, ρ<0.05; 0.05% IL-2^+^TNF-α^+^ cells to 0.16% post-challenge, ρ<0.001; and 0.19% TNF-α^+^ cells to 0.34%, ρ<0.05, pre- and post-challenge, respectively (Figure 7A).

These increases were substantially greater in the lungs following challenge:

0.1% IFN-γ^+^IL-2^+^TNF-α^+^ increases to 3.84%; IFN-γ^+^TNF-α^+^ 0.16 to 2.26%; IL-2^+^/TNF-α^+^ 0.06 to 4.6% and TNF-α^+^ 0.38 to 6.01%, ρ<0.05 (Figure 7B). Multifunctional responses induced by challenge of naïve animals were less than induced by BCG vaccination (Figure 7). CD8 T cell responses in both spleen and lung were negligible (data not shown). Unless stated, changes form pre-to post-challenge were not statistically significant (n.s.).

Together these data demonstrate the preferential expansion of cells expressing two or more cytokines, with the multifunctional lung lymphocytes exhibiting a mean 38 fold expansion. IFN-γ/TNF-α double positive and IFN-γ/IL-2/TNF-α triple positive cells were the functional phenotypes most strongly associated with protection. Interestingly, infection induced a substantial expansion of TNF-α single positive cells and IL-2/TNF-α double positive cells.

### BCG induces a CD4^+^CD44^hi^ multifunctional T cell response for at least 18 months post vaccination

**Figure 8:**
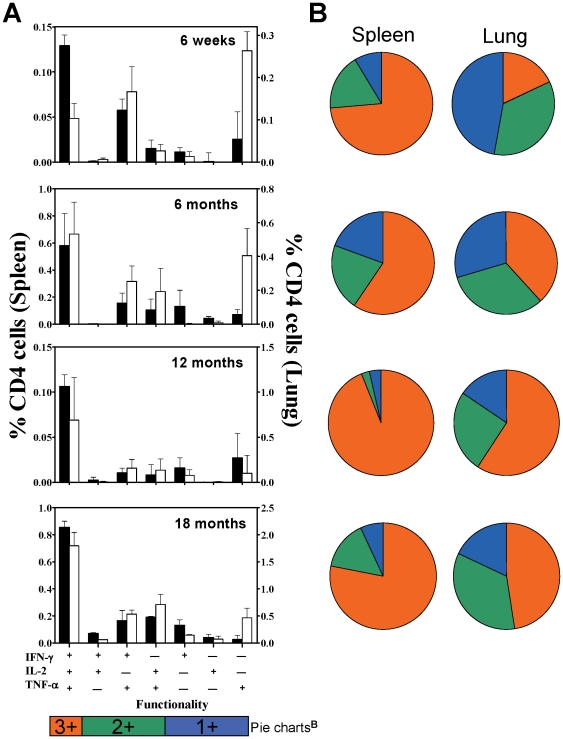
BCG induced CD4^+^CD44^hi^ multifunctional T cells persist at least 18 months post vaccination. The frequencies of antigen-specific CD4^+^CD44^hi^ cells exhibiting all functional phenotypes of IFN-γ, IL-2 and TNF-α production were determined by ICS in the spleen (black bars) and lungs (white bars) of BCG immunized mice up to 18 months post immunization (A). Bars represent mean (±SE) frequency of these cells as a % of total CD4^+^ cells minus the responses of age matched sham control mice, n = 2–8. Pie charts (B) represent the proportion of these cells exhibiting a 1^+^, 2^+^ or 3^+^ cytokine production phenotype, at the corresponding time points.

To assess the longevity of the multifunctional CD4 T cell response, spleen and lung lymphocytes from BCG immunised mice were isolated, restimulated with antigen cocktail and interrogated by ICS at, 1, 6, 12 and 18 months post immunization (Figure 8). Both the frequency (Figure 8A) and absolute proportion (Figure 8B) of IFN-γ^+^IL-2^+^TNF-α^+^ cells increased over time in both spleen and lungs, but the lung maintained a higher proportion of double cytokine producers, IFN-γ^+^TNF-α^+^ and IL-2^+^TNF-α^+^, (Figure 8B).

There was a clear biphasic nature in the magnitude of the antigen-specific CD4 T cell response in both the spleen and lung (Figure 8A). At 12 months post vaccination the frequency of the CD4^+^ multifunctional T cells dropped to the levels observed at 6 weeks post-vaccination, but at 18 months had risen equivalent to those observed at 6 months. The proportion of multifunctional responders remained similar at this time (Figure 8B). This was repeatedly observed in several separate experiments, but the mechanisms underlying this are unclear at this time, and beyond the scope of this study.

These data demonstrate that BCG immunization induces a complex T cell response which persists at least 18 months post vaccination. We also determined whether BCG vaccine induced immunity persisted in parallel with multifunctional T cells by challenging mice 12 months post-vaccination, i.e. at a time of stable multifunctional T cell responses. As shown in Table 1, protection at one year remained comparably significant to mice challenged six weeks following BCG immunization.

## Discussion

To more closely understand the T cell mechanisms underlying BCG vaccination induced immunity would help to identify immune correlates of protection and facilitate the rational design of improved replacement or adjunct tuberculosis vaccines. Given the established central role of CD4 T cells in BCG induced immunity, we sought to characterise the generation of memory CD4 T cell responses to BCG vaccination and the response of these cells during the active expression of protection against virulent mycobacterial challenge in a murine model.

We demonstrate that a single systemic BCG immunization induces both systemic and mucosal multifunctional CD4 T cells with a surface phenotype associated with highly differentiated effector memory T cells (T_EM_). These T_EM_ were detectable for at least 18 months post vaccination, underwent significant expansion in the lungs following infection and are, therefore, strongly associated with protection against *M. bovis* challenge.

Assessing the magnitude and frequency of pivotal cytokines, such as IFN-γ has remained the standard measurement of vaccine induced T cell memory for many years. It has become clear recently, however, that these relatively simple *ex vivo* assays cannot consistently predict vaccine success or adequately describe the complexity of the CD4 T cell response to vaccination [6]. Indeed, these assays must be interpreted with care as it is well documented that ESAT-6 and PPD stimulated IFN-γ correlate with bacterial load [12], [26], [27], whilst TB10.3 and TB10.4 induced responses strongly correlate with protection [9], [11]. In the current study, we have therefore used a defined cocktail of secreted, strongly antigenic proteins to circumvent this bias toward a single antigen, or ill defined crude preparation skewing the results.

Recent reports have identified and highlighted the role of multifunctional T cells in a number of disease and vaccination models [15], [18], [19], [20], [28]. Multifunctional CD4 T cells define a correlate of protection against *Leishmania major* [17] and associate with vaccine induced protection against *M. tuberculosis* in mice [18], [19], [29], non-human primates (NHP) [20] and vaccine induced responses in humans [21], [30], [31]. Multifunctional CD8 T cells associate with HIV non-progressors [15]. Protective immunity in the lungs of influenza infected mice is also characterized by CD4 and CD8 T cells with a multi-functional phenotype [16]. In contrast, recent reports correlate *M. tuberculosis* associated multifunctional cells with active disease in humans, [22], [23], [24]. Whether multifunctional cells represent a non-protective profile in active human disease or their protective role is mediated by other factors in this more complex scenario remains to be elucidated.

Our study however, is the first to describe significant expansion of BCG-specific lung resident multifunctional CD4 T_EM_ cells during the protective response to virulent mycobacterial challenge.

Supported by the analysis of the cells identified in our study, A common feature of multifunctional T cells, [17], [18], [19], is the ability to produce more cytokine than single positive cells, on a per cell basis, termed a higher ‘quality’ of response, allowing concomitant effector function (reviewed in [32]).

There are conflicting reports in the literature as to whether vaccination induced multifunctional T cells represent a subset of effector (T_EM_) or central (T_CM_) memory T cells. Darrah *et al.*, describe Leishmania vaccine multifunctional T cells to predominantly associate with a T_EM_ phenotype [17], whilst Lindestrom *et al.*, identify tuberculosis subunit vaccine induced multifunctional cells to be T_CM_ [19], both studies using CCR7 expression as the defining factor.

Our results suggest that the multifunctional CD4 T cells defined in this study express a CD44^hi^CD62L^lo^CCR7^−^ phenotype, which in concordance with Darrah *et al.*, [17] we propose to be T_EM_ cells. Another, recent report supports this conclusion; observing a predominant T effector (T_Eff_) or T_EM_ cell phenotype in the lungs of BCG vaccinated or *M. tuberculosis* infected mice [33], although cytokine multifunctionality was not established in that study.

Interestingly; Orme [34] hypothesises that development of T_CM_ from effector responses are impaired by persisting mycobacteria following infection or BCG vaccination. This is supported by published data [35], [36], [37] and our own recent observations that parenteral BCG vaccination results in BCG bacilli persisting, albeit at low bacillary numbers, for at least sixteen months post-vaccination in spleen and lymph nodes (Kaveh and Hogarth manuscript in preparation). These observations therefore suggest that the detection of multifunctional CD4 T_EM_ rather than T_CM_, may be due to persistence of antigen in sufficient quantity to support the maintenance of T_Eff_ and T_EM_ cells. In contrast, the development of T_CM_ responses may be more dependent on the clearance of BCG and consequently lower antigen availability, consistent with the hypothesis that T_CM_ cells are generated once antigen is cleared. Whilst this is a strongly held hypothesis, the effect of continued BCG derived antigen on the maintenance of this T_EM_ population needs to be demonstrated experimentally, for which experiments are underway in our laboratory.

CD27^−^ CD4 T cells have been identified as protective against tuberculosis via IFN-γ production, although simultaneous cytokine production potential was not assessed in these studies [38], [39]. Loss of CD27 is associated with the development of effector function [38], [40], [41], [42]. Whilst CD27^−^ CD4 T cells are considered short lived terminally differentiated effector (T_EFF_ or T_EM_) cells in some experimental systems [39], [43], the concomitant production of IL-2 and IFN-γ by these cells seen here, infers memory as well as effector function as suggested by others [41]. We, therefore, propose that the multifunctional CD44^hi^CD62L^lo^CCR7^−^CD27^−^ CD4 T cells we observe represent a population of highly differentiated, but not terminally differentiated T_EM_ cells. The ability of these T_EM_ to persist in the lung for at least eighteen months post vaccination is striking. This may reflect persistence of antigen providing prolonged low level stimulation promoting survival as discussed; or perhaps, continuing priming by persistent BCG providing recent immigrant T_EM_ to the mucosal T cell pool. As mentioned, current experiments investigating the effect of BCG persistence are aimed at clarifying the mechanism following BCG vaccination.

In the current study, the antigen specific CD4 T cells most strongly associated with BCG vaccination were of an IFN-γ^+^IL-2^+^TNF-α^+^ and IFN-γ^+^ TNF-α^+^ phenotype and were seen to expand on an *M. bovis* challenge. However, the expansion of the TNF-α^+^ and IL-2^+^TNF-α^+^ cells may also reflect an important role during protection. In support of these findings, the former two cell subsets dominated in other studies of leishmania and BCG vaccine responses [17], [18], [19], [44]. These include studies evaluating protective subunit TB vaccines, which additionally induced a substantial population of the IL-2^+^TNF-α^+^ subset. A greater importance of the IFN-γ/IL-2/TNF-α triple producers may be suggested by our data revealing a decrease in the proportional representation of the IFN-γ/TNF-α double producers from 6 months post vaccination onwards. With cohorts of mice *M. bovis* challenged at 6 weeks or 12 months post vaccination displaying comparative log_10_ protection, the single common protective correlate was a high frequency of BCG specific IFN-γ/IL-2/TNF-α triple producing CD4 T cells.

A recent study by Mu *et al*. [45] using intranasal vaccination of mice with a protective adenovirus vaccine demonstrated that persistent antigen-specific CD8 T cells also displayed an activated T_EM_ phenotype. Further, these cells are self-renewable in an antigen-dependent manner, and could be maintained independent of peripheral T cell supply. It is therefore plausible that the expansion of multifunctional T_EFF_/T_EM_ cells following *M. bovis* infection in the present study is due to expansion of cells resident in the lung at the time of infection. However, we can not rule out that these cells are ingressing from draining lymph nodes or other peripheral lymphatic tissues following infection, as we observed identical T cell populations in the spleen.

We, therefore, investigated a number of potential lung homing markers including the heterodimer α4β1, the activation marker CD11a and the chemokine receptor CXCR3. The expression of α4β1 has been described to localize *M. tuberculosis-*specific Th1 responses in human lungs [46], promote lung-homing of CD4 T cells in asthmatic patients [47], whilst blocking α4 integrin and VCAM-1 significantly inhibits the migration of memory T cells to bronchiolar associated lymphoid tissue (BALT) in mice [48]. The integrin CD11a is required for host resistance following infection with *M. tuberculosis*. In CD11a^−/−^ mice, antigen-specific T cell priming is delayed and T cell trafficking to the lung is impaired [49]. A deficiency in CXCR3, the receptor for the IFN-γ inducible T cell chemokines CXCL9, CXCL10 and CXCL11, impairs the trafficking of adoptively transferred Th1 cells to the lung in wild-type mice [50]. Expression of CXCR3 ligands are abundant in granulomas of *M. tuberculosis* infected Macaques and promote the recruitment of CXCR3^+^ cells [51]. The expression of CXCL9, CXCL10, and CXCL11 were also highly increased in BCG-vaccinated mice after *M. tuberculosis* infection [52].

Together, our data showing the up-regulation of these homing markers on a significant proportion of the BCG induced spleen derived multifunctional cells, infers these may be a source of the cells detected in the lung. The down-regulation of these markers on the lung derived cells suggests their expression may no longer be required once the cells are resident in the lung.

Interestingly, preliminary data from our laboratory indicates that sorted spleen derived CD44^hi^ CD4 T cells home and accumulate in the lungs but not spleen following adoptive transfer and *M. bovis* challenge. This confirms that these cells possess lung homing capability under inflammatory conditions (data not shown).

The study by Mu *et al*. and earlier reports by this group is also interesting in that they correlated protection after intranasal viral vaccination with the presence of antigen-specific CD8 T cells in the airway lumen and not in the lung interstitium. In contrast we found only minor CD8 responses, and the described multifunctional CD4 T cell populations in our study were mainly located in the interstitial tissue of the lung rather than the lumen. The differences could be due to the nature of the adenoviral vector system favouring CD8 responses and the intranasal challenge route, respectively.

Our data support the hypothesis that these multifunctional T_EM_ CD4 T cells correlate with protective immunity, and indeed could constitute the protective T cell sub-population. This is clearly supported by our observation of a dramatic expansion of these cells following mycobacterial challenge. However, we acknowledge that this hypothesis will need to be confirmed in further studies including adoptive transfer and *in vitro* depletion experiments, which are underway.

The preferential induction of a mucosal response by a non-mucosally administered BCG demonstrated in the present study was striking. Other reports of a strong lung response to immunization involve intranasal vaccine delivery [18], [53], [54]. These results thus demonstrate the potential of systemic vaccination to induce long lasting immunity and resident specific T cells in the lung. This is an encouraging observation and could explain why intranasal viral subunit boosting is effective in boosting systemically primed BCG responses.

Our data demonstrate that a persistent mucosal population of T_EM_ cells can be induced by parenteral immunization, a feature only previously associated with mucosal immunization routes; and that these multifunctional T_EM_ cells are strongly associated with protection. We propose that these cells mediate protective immunity, and that vaccines designed to increase the number of relevant antigen-specific T_EM_ in the lung may represent a new generation of TB vaccines.

In conclusion, the major observation in our study is that multifunctional memory T cells can be induced and maintained in the lung for a long period of time after systemic BCG vaccination without the need to use a mucosal route.

## Materials and Methods

### Ethics

All animal work was carried out in accordance with the UK Animal (Scientific Procedures) Act 1986; under appropriate personal and project licences. The study protocol was approved by the AHVLA Animal Use Ethics Committee (UK Home Office PCD number 70/6905).

### Animals

Female BALB/c mice were obtained from SPF facilities at Charles River UK Ltd and used at 8 weeks of age. All animals were housed in appropriate BSL3 containment facilities at AHVLA.

### Mycobacteria

The vaccination strain used was the human vaccine *M. bovis* BCG Danish 1331, prepared as per manufacturer's instructions (SSI, Denmark).

*Mycobacterium bovis* isolate AF2122/97 was grown to mid-log phase in Middlebrook 7H9 broth (Gibco, UK) supplemented with 4.16 g/L pyruvic acid, 10% v/v OADC and 0.05% v/v Tween 80 (all Sigma, UK), subsequently frozen at −80°C, and used for all virulent challenges.

### Mycobacterial antigens

M7, a pool of 7 secreted, immunogenic recombinant mycobacterial proteins (Rv1886c, Rv0251, Rv0287, Rv0288, Rv3019c, Rv3763, Rv3804c) was used for antigen-specific stimulation. Rv1886, Rv3019c Rv3804c and Rv3763 were purchased from Lionex GmbH, Germany. Rv0251, Rv0287 and Rv0288 were purchased from Proteix s.r.o., Czech Republic.

### Immunization and mycobacterial challenge

Mice were immunized with a single intradermal (i.d.) injection of 2×10^5^ CFU of BCG. Control mice were immunized with PBS. Six weeks following immunization, mice were challenged intranasally with approx. 600 CFU of virulent *M. bovis* as previously described (Logan et al, 2008). Four weeks after challenge, the mice were euthanized and the lungs and spleens removed and homogenised. These homogenates were serially diluted and plated out on modified Middlebrook 7H11 agar media [55]. Bacterial colonies were enumerated four weeks later following incubation at 37°C. For protection longevity study, mice were immunized with BCG or PBS at week zero or week forty eight. At week fifty four all mice were challenged with approx. 600 CFU of virulent *M. bovis*. Four weeks after challenge bacterial load in spleen and lungs were assessed as previously. For long term BCG induced immunology, spleen and lungs were harvested as described at up to eighteen months post vaccination.

### Cell isolations and stimulations

To isolate cells from different organs, the following procedure was undertaken in sequence.

Bronchoalveolar lavage (BAL) cells were isolated as described previously [54]. Briefly, the lungs were removed, catheterised and flushed five times with HBSS. BAL derived cells were resuspended at 5×10^6^/ml for assays. The right ventricle was then perfused with ice cold HBSS prior to aseptic removal of the lungs and spleen.

Spleen cells were prepared by passage through a 40 µm cell strainer into DMEM (Sigma, UK) supplemented with 10% v/v FCS and antibiotics (100 U/ml penicillin and 100 µg/ml streptomycin) (Gibco). Following washing at 300 g for 8 min, cells were re-suspended at 5×10^6^/ml for assays.

Lung interstitial cells were isolated by finely minced the lungs, and re-suspended into 25 ml digestion media (supplemented DMEM plus 150 U/ml Collagenase I (Gibco) and 10 U/ml DNAse II (Sigma). Following stirring (∼200 rpm) at 37°C for 1 hour, lung cells were passed through a 40 µm cell strainer, washed & re-suspended at 5×10^6^/ml (5×10^5^ post challenge) for assays.

Cells were cultured with a pool of 7 recombinant antigens as described, each antigen at a final concentration of 2 µg/ml for all assays.

### ELISPOT

5×10^5^ cells (or 5×10^4^ lung cells post challenge) were incubated in duplicate in 96 well filter plates (MSIPS4510 Millipore, Ireland) with or without antigen for 16 hours and the frequency of IFN-γ secretors detected by ELISPOT (Mabtech, Sweden), as per manufacturer's instructions

### Flow cytometry

Cells isolated from spleen or lungs were stimulated with recombinant antigen pool and 1 µg/ml anti-CD28 (BD Biosciences, UK) for 2 hours at 37°C/5% CO_2_. Brefeldin A (Sigma) was added at 10 µg/ml and cells cultured for a further 16 hours. Cells were washed (300 g/5 mins) and surface stained with CD27 - PE, CD4 - PE-TexasRed (Invitrogen, USA) or – APC-H7, CD44 - PE-Cy5 or - Qdot705 (Invitrogen, USA), CD62L – biotin, - FITC, – PE or – PE-TexasRed (Invitrogen), CD8 - APC-Cy7, Violet fixable dead cell stain and streptavidin - Pacific Orange (both Invitrogen). Subsequently the cells were washed, treated with BD Biosciences Cytofix/Cytoperm according to manufacturer's instructions and stained intracellularly with IFN-γ - APC, IL-2 – FITC, - PE or - PE-Cy7 and TNF-α - PE-Cy7 or - FITC (eBioscience, USA). All antibody conjugates were purchase from BD Bioscences except where stated). Following washing, cells were analysed using a CyAn ADP analyser and Summit software (Beckman Coulter, USA). All analyses and plots were gated on a minimum of 100 000 live lymphocytes.

### Statistical analyses

All data was analysed using GraphPad Instat 3 statistical package (GraphPad, USA). Mycobacterial counts were log_10_ transformed and analyzed using the unpaired Student's *t*-test (Exp. 1), or ANOVA with Bonferroni post-test (Exp. 2). ELISPOT data were analysed using the unpaired Student's *t*-test with Welch correction.
